# Modelling Mass Casualty Decontamination Systems Informed by Field Exercise Data 

**DOI:** 10.3390/ijerph9103685

**Published:** 2012-10-16

**Authors:** Joseph R. Egan, Richard Amlôt

**Affiliations:** 1 Microbial Risk Assessment, Emergency Response Department, Health Protection Agency, Porton Down, Salisbury, Wiltshire SP4 0JG, UK; 2 Behavioural Science, Emergency Response Department, Health Protection Agency, Porton Down, Salisbury, Wiltshire, SP4 0JG, UK; Email: richard.amlot@hpa.org.uk

**Keywords:** chemical hazard release, mass casualty incidents, decontamination, computer simulation, operations research.

## Abstract

In the event of a large-scale chemical release in the UK decontamination of ambulant casualties would be undertaken by the Fire and Rescue Service (FRS). The aim of this study was to track the movement of volunteer casualties at two mass decontamination field exercises using passive Radio Frequency Identification tags and detection mats that were placed at pre-defined locations. The exercise data were then used to inform a computer model of the FRS component of the mass decontamination process. Having removed all clothing and having showered, the re-dressing (termed *re-robing*) of casualties was found to be a bottleneck in the mass decontamination process during both exercises. Computer simulations showed that increasing the capacity of each lane of the re-robe section to accommodate 10 rather than five casualties would be optimal in general, but that a capacity of 15 might be required to accommodate vulnerable individuals. If the duration of the shower was decreased from three minutes to one minute then a per lane re-robe capacity of 20 might be necessary to maximise the throughput of casualties. In conclusion, one practical enhancement to the FRS response may be to provide at least one additional re-robe section per mass decontamination unit.

## 1. Introduction

During the morning rush-hour of 20 March 1995, terrorists released the nerve agent sarin within 5 separate commuter-trains on the Tokyo subway system, exposing 15 stations to the toxic chemical in the minutes that followed [1]. The attack resulted in 12 fatalities and approximately 5,500 people became ill. Nine months prior to the Tokyo attack the organisation responsible, Aum Shinrikyo, had also attacked a central area of Matsumoto city, Japan, by releasing sarin gas from an over-ground truck [2]. Approximately 600 people were affected by the noxious cloud, including seven who died. Prior to the Japanese sarin attacks chemical agents were more generally considered as military weapons that had been deployed in a number of twentieth century wars. However, the events of 9/11, 7/7 and other terrorist attacks of the early twenty-first century brought sharply into focus that Western cities might experience other chemical attacks and that further planning for such incidents was a public health priority. Indeed, the threat of exposure to chemical agents is not limited to a deliberate release scenario. On 10 July 1976, there was an accidental chemical release from a manufacturing plant near the town of Seveso, Italy. People who happened to be in the path of the cloud developed headaches and eye-irritation, and a few children were admitted to local hospitals with lesions on exposed skin [3,4]. Eight years later a large gas leak from a pesticide plant in Bhopal, India killed at least 3,800 local people in the hours that followed [5]. More than half a million people were exposed to the toxic chemicals; approximately 120,000 of them continue to suffer from a variety of chronic disorders [6]. It is clear from the differing magnitudes of these various incidents that public health authorities need to be prepared to mitigate both small and large-scale chemical releases.

In the event of either a deliberate or accidental chemical release contaminated individuals would require rapid decontamination to reduce their probability of illness or death. To prepare for such an incident the emergency services perform field exercises using volunteer “casualty” actors. While these exercises can be extremely useful for testing responses and can help to identify important lessons for the future [7] they are highly resource intensive to stage. One partial solution to this problem is the use of computer simulation via operational research techniques. Indeed, large-scale exercises in the US have been complemented by mathematical models of antibiotic distribution centres [8] and mass vaccination clinics [9] with the aim of optimising staffing levels. In the UK computer simulations of mass decontamination systems have recently been performed by Albores and Shaw [10], who took a holistic approach that aimed to investigate resource requirements (vehicles, equipment and staff) necessary to decontaminate *x* casualties in *y* hours. Their model was informed by a number of interviews with key stakeholders who were knowledgeable of the mass decontamination process and/or had exercise experience. In order to build on their study we collected casualty movement data during two field exercises and subsequently modelled some more fundamental aspects of the mass decontamination process, both of which had not been explicitly addressed in earlier work.

## 2. Background

In the UK mass decontamination of the “walking wounded” (termed *ambulant casualties*) would be undertaken by the Fire and Rescue Service (FRS) on behalf of the Ambulance Service as a result of the New Dimension programme [11]. Ambulant casualties would be required to move away from the area of highest contamination (termed *Hot Zone*) to an area ideally up-wind of the incident scene (termed *Warm Zone*). If deemed necessary ambulant casualties may undergo either emergency decontamination or interim mass decontamination. The former process involves simply the application of water to ambulant casualties via, for example, building sprinklers or FRS hoses. The latter process is more structured and involves ambulant casualties walking through a makeshift shower utilising FRS hoses suspended from ladders fitted between parallel fire engines. Ambulant casualties would then be directed to a “casualty collection point” from where the FRS would subsequently conduct mass decontamination procedures (as described further below). 

**Figure 1:**
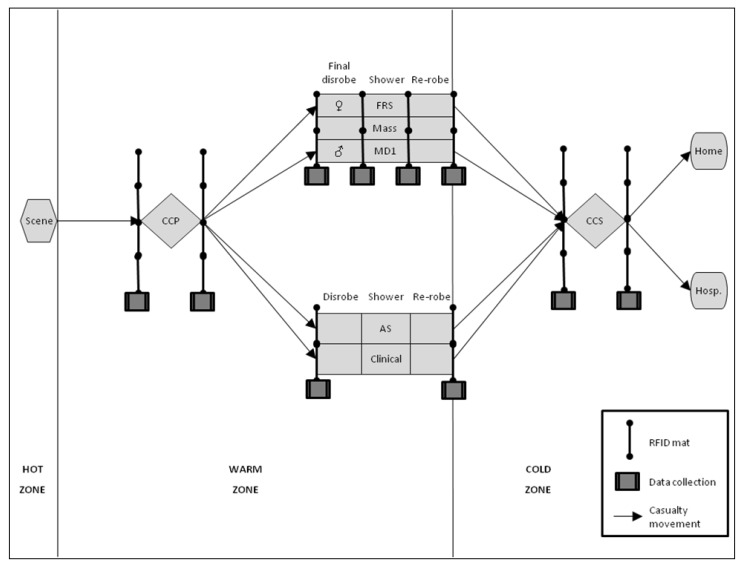
Schematic of casualty movement through an idealised mass decontamination response. The Police Service inner cordon runs around the Hot and Warm Zones. Emergency decontamination/interim mass decontamination for ambulant casualties may take place between the Scene and the casualty collection point (CCP). Ambulant casualties progress to the Fire and Rescue Service (FRS) mass decontamination unit (termed *MD1*) in groups of five males and five females; FRS personnel patrol the middle lane removing disrobe clothing and providing re-robe packs. Non-ambulant casualties progress to the Ambulance Service (AS) clinical decontamination unit; two lanes are available to decontaminate individual casualties. The middle lane of the MD1 can also support clinical decontamination should it be needed but only when specifically configured and not simultaneously with mass decontamination. All casualties finally enter the casualty collection station (CCS) and are assessed for hospital (Hosp.) treatment or otherwise (Home). Radio Frequency Identification (RFID) detection mats were placed at shown locations in Exercise 1. The lights of the MD1 flow control system are located above the four RFID detection mats positioned at the MD1.

Those individuals who are unable to walk (termed *non-ambulant casualties*), either because they are symptomatic, seriously injured in any initial explosion/accident or through a pre-existing disability, would be triaged by the Hazardous Area Response Team (HART) of the Ambulance Service [12]. Non-ambulant casualties would then be conveyed out of the Hot Zone by the FRS and individually decontaminated in the Warm Zone by the Specialist Operational Response Team (SORT) of the Ambulance Service (note that HART does not apply in Scotland with SORT performing both roles). If the release was considered to have been deliberate then the Police Service would seek to maintain an “inner cordon” around the Hot and Warm Zones, in an attempt to apprehend the perpetrators and collect crime-scene evidence. Having passed through the decontamination process all casualties would then move to a “casualty clearing station” located in an area outside of the inner cordon (termed *Cold Zone*) where they would be re-triaged for further hospital treatment if required. Depending on the circumstances of the chemical release and the health status of the casualties, the Police Service may perform brief information gathering interviews at this stage. Figure 1 provides a schematic of the casualty movement through an idealised mass decontamination response.

In recent years mass decontamination procedures have developed as a result of training and operational experience, and have also been informed by the work of Albores and Shaw [10]. In their study ambulant casualties are expected to remove their clothes in the disrobe section of the FRS mass decontamination unit (termed MD1—see Figure 1) before showering in the middle section and then drying and dressing with fresh clothes in the re-robe section. Although groups of five males and five females are still required to enter two separate lanes of the MD1, the original batch disrobing has been replaced by an initial mass disrobe at the casualty collection point (*i.e.*, outside of the MD1). Thus, ambulant casualties are now required to remove their potentially contaminated clothing and are provided with temporary clothing from a disrobe pack to maintain warmth and preserve their modesty whilst awaiting MD1 construction. The original disrobe section of the MD1 is now used as a final disrobe section where ambulant casualties remove their disrobe clothing prior to entering the shower section.

The other major change is the introduction of an automated flow control system to the MD1. Lights are located at the entrance to the final disrobe, shower and re-robe sections as well as the exit of the re-robe section.

For three minutes a red “Wait” pictogram is illuminated during which time the boiler provides two minutes of soapy water for washing in the shower section followed by one minute of water for rinsing. The lights then switch to illuminate a green “Enter” pictogram for fifteen seconds allowing casualties to move through to the next section (or enter/leave the MD1). An audible prompt also accompanies this stage following which the green “Enter” pictogram then flashes for a further five seconds to indicate that the cycle is about to revert back to the red “Wait” pictogram. Having entered the MD1 the ideal situation is for two groups of five (fe)male ambulant casualties to pass through the entire structure in 10 minutes, having spent three minutes in each section with 20 second transitions between each section and exiting the MD1.

## 3. Field Exercise Data

To evaluate the current procedures outlined above we collected casualty movement data from two UK mass decontamination field exercises. Exercise 1 assumed an accidental release of a hazardous material and therefore involved the local FRS and Ambulance Service. There were 50 casualties in total comprising both able-bodied and disabled volunteers who were recruited mainly from a university local to the exercise location and a number of disability organisations. The casualties had a median age of 24 (range 16 to 67) and a standard deviation of 13.4 years. Exercise 2 assumed a deliberate release and therefore also involved the Police Service. A total of 130 volunteer casualties comprised only the able-bodied who were recruited mainly from two further-education colleges local to the exercise location. The casualties had a median age of 17 (range 9 to 53) and a standard deviation of 6.6 years. For both exercises the characteristics of the casualties (e.g., age, gender, disability) and their total number were largely determined in advance by the exercise organisers and were outside of the authors’ control. Casualties were tracked by providing each of them with a passive Radio Frequency Identification (RFID) tag supplied by FR Systems (Stoke on Trent, UK). Mats that could detect the presence of the RFID tags were placed at pre-defined locations (see Figure 1) allowing a calculation of the time that each casualty had spent at each stage of the decontamination process. For the purposes of this study we restricted our analysis to the processing times of ambulant casualties within the three sections of the MD1. Interested readers can find further details describing the casualty movement analysis in the Supplementary Material.

**Figure 2:**
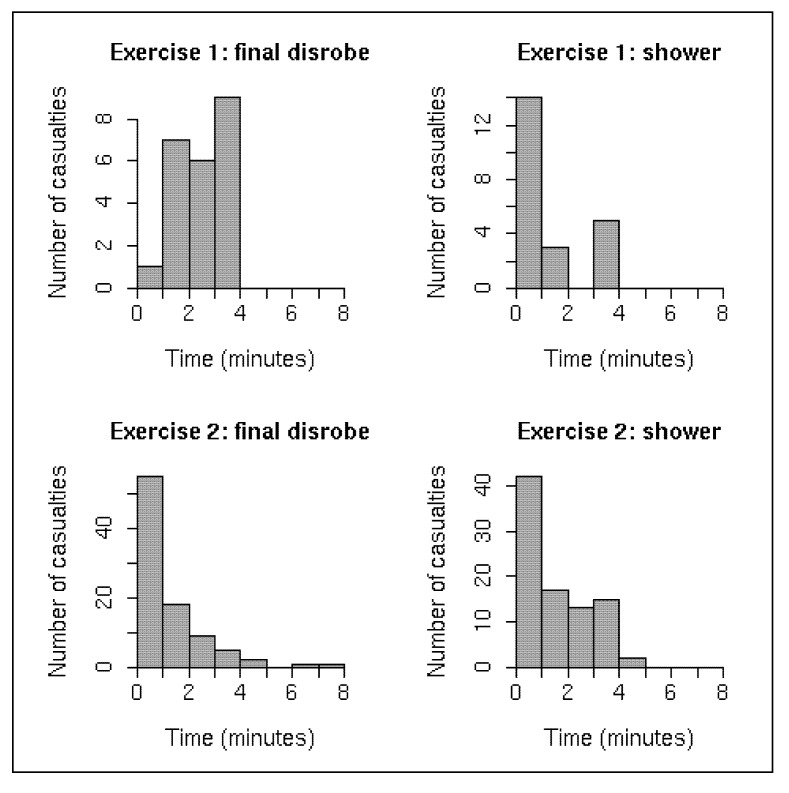
Histograms of the final disrobe and shower durations for both exercises.

**Figure 3:**
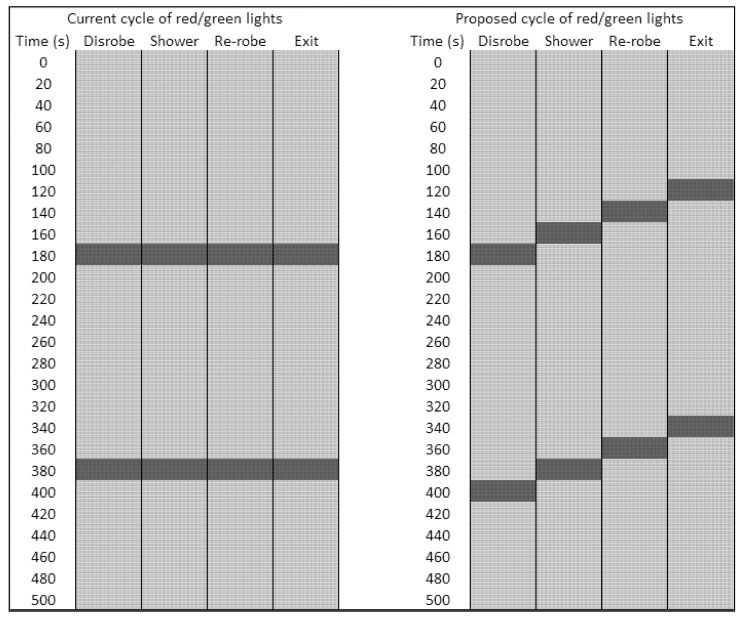
Current (**left**) and proposed (**right**) flow control system. The light and dark grey sections represent the time when the lights are illuminated red and green, respectively. “Disrobe” refers to the final disrobe section as described in the main text.

Figure 2 shows a range of times spent in each section of the MD1 suggesting that the flow control system was often ignored or misunderstood by casualties during both exercises. Notably, casualties in both exercises walked through the shower section into the re-robe section without stopping. This behaviour has also been anecdotally observed at other field exercises but the data provided in Figure 2 importantly provides quantitative evidence. One possible explanation for this occurrence is that because all of the flow control system lights turn green at the same time, casualties simply pass by each light in the same way as they would in a vehicle travelling through multiple sets of traffic lights. Observational data collected during each of the two exercises (not reported here) suggests that this issue may occur particularly when the number of casualties in each section of the MD1 is fewer than the maximum capacity, and if verbal instructions from emergency services personnel to casualties are not heard or fully understood [13]. A technical solution to this problem might be to stagger the traffic lights so that having entered one section on a green light, the light to the next section would have already changed to red, thus instructing casualties to wait before proceeding (see Figure 3). Better communication between emergency services personnel and casualties might also help to improve the flow of casualties through the MD1.

**Figure 4:**
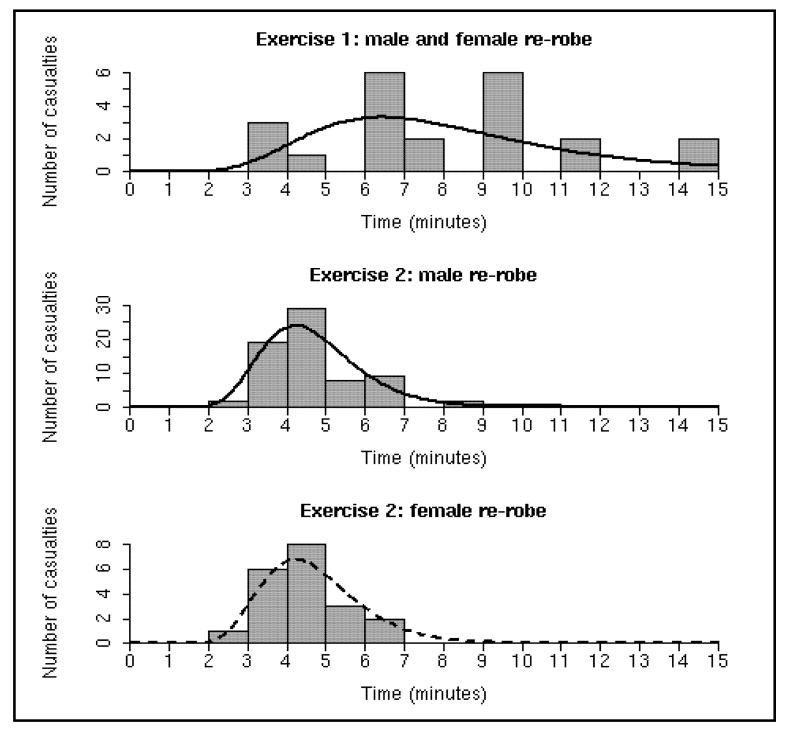
Histograms of the re-robe durations for both exercises. The solid black lines show the baseline (**middle**) and worst-case (**top**) re-robe distribution fits to the data. The dashed black line overlays the baseline fit on the female data in order to highlight their similarity. The worst-case fit is based on a combination of male and female data from Exercise 1 whereas the baseline fit is based only on the male data from Exercise 2.

Let us assume that the enhancements outlined above are made to the flow control system and/or that all casualties correctly understand how to proceed through the MD1. In this situation the final disrobe section would essentially become a waiting area because the process of removing the disrobe clothing would likely take significantly less time than the allocated three minutes. Similarly the process of showering could be considered complete when the lights change from red to green after three minutes. However, re-robing is particularly dependent on the individual casualty, *i.e.*, the re-robe data shown in Figure 4 are less likely to be an artefact of the flow control system but more likely to reflect the actual time that it takes to dry and dress. Therefore, given that many casualties require more than the allotted three minutes to re-robe (see Figure 4), it is immediately clear that the re-robe section is a potential bottleneck in the MD1. According to system improvement philosophies the best way to increase throughput is to increase the capacity and/or decrease process time at bottlenecks [9]. Although it may be possible to speed up re-robing times by making alterations to the clothing within the re-robe pack, in this study we investigate varying the capacity of the re-robe section via computer (discrete event) simulation. In addition, controlled volunteer trials (rather than field exercises) have previously shown that a doubling of the showering duration has demonstrated no significant improvement to the efficacy of decontamination [14]. Therefore, it is conceivable that there may be scope for decreasing the shower duration without reducing its effectiveness which could help to increase the rate at which casualties flow through the system.

## 4. Methods

Although analytical techniques could have been investigated we were concerned that model formulation and development might quickly become intractable. Therefore, we took a more flexible approach and built a simple model describing the MD1 using the SIMUL8^® ^software. The final disrobe, shower and re-robe sections were modelled as work-stations with work items representing casualties. In addition to the “drag and drop” functionality used to construct the basic model, we wrote Visual Logic code to capture the flow control system. Casualties could only enter/exit the system and transition between sections when the lights were green and when there was available capacity to do so. In order to capture the behaviour of the system we simulated an unlimited number of casualties queuing at the entrance to the MD1. We also set a conservative warm-up period of 100 minutes to allow the initial simulated casualties to filter through the three MD1 sections before collecting 5,000 additional minutes of simulation output. Note that the computer run-time was not meant to reflect an actual response scenario but rather to allow for a robust statistical analysis of the results. Processing times for the final disrobe and shower sections were set at 3 minutes, equal to the duration of the red light. Any lower values would have produced the same results because the lights are the controlling factor here. Re-robing times were modelled on the raw data collected during the two exercises. Two-parameter gamma, log-normal, and Weibull probability density functions (PDFs) were fitted to the data using the “mle” function in the R stats4 package [15]. As a sensitivity analysis we varied the simulated shower duration between one and four minutes in one minute intervals by varying the duration of the red lights by the same values. The duration of the green lights remained unchanged at 20 seconds to allow the transition between sections as before. We assumed that the final disrobe could still be completed within one minute (equivalent to the shortest considered shower duration) and so this section effectively remained as a waiting area. 

## 5. Results

### 5.1. Parameterisation

Using the 71 male re-robing times captured from Exercise 2 the maximised log-likelihood for the gamma, log-normal, and Weibull PDFs were −119.12, −116.55, and −128.72, respectively. Therefore, on the basis of the models that were analysed, the best fit to the data was provided by the log-normal PDF with parameters µ = 1.51 (95% confidence interval = 1.45–1.58) and σ = 0.27 (95% confidence interval = 0.24–0.33), termed *baseline re-robe distribution* (see Figure 4, middle panel). A χ^2^ goodness-of-fit test confirmed the quality of the fit (*P *= 0.30, two degrees of freedom). We also found that a Kolmogorov-Smirnov test revealed no evidence against the 20 female re-robing times being drawn from the baseline re-robe distribution (*P *= 0.77), suggesting that males and females generally take the same amount of time to re-robe (Figure 4, bottom panel).

In Exercise 1 there were only 10 male and 13 female casualties that were tracked through the MD1. Due to the small sample size and because the data from Exercise 2 suggested that there were no significant differences between male and female re-robe times we combined the male and female data. The maximised log-likelihood of the log-normal PDF (−55.22) was greater than that of the gamma (−54.97) and equal to that of the Weibull. We chose the log-normal PDF with parameters µ = 2.02 (95% confidence interval = 1.85–2.19) and σ = 0.40 (95% confidence interval = 0.30–0.55), termed *worst-case re-robe distribution* (see Figure 4, top panel), because the log-normal PDF had been shown to be superior with the larger sample size in Exercise 2. Again, a χ^2^ goodness-of-fit test confirmed the quality of the fit (*P *= 0.29, one degree of freedom).

### 5.2. Simulation

**Figure 5:**
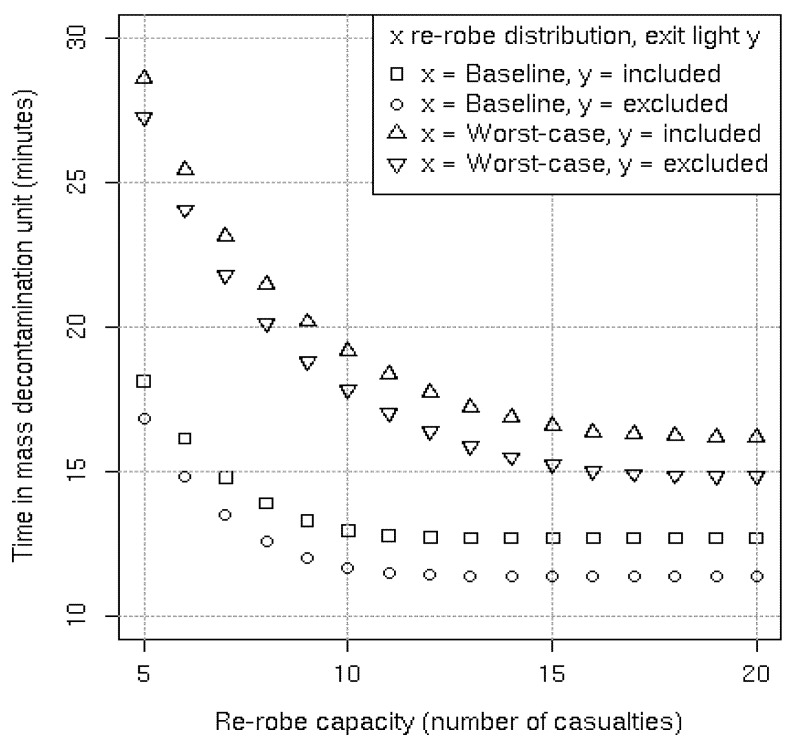
Average simulated results per lane with a three minute shower duration.

The following results refer to a single lane of the MD1 and are applicable to groups of males and females alike. Figure 5 shows that with the baseline re-robe distribution the average simulated time in the MD1 is 17.7 minutes, almost eight minutes longer than the theoretical minimum of 10 minutes. Incrementally increasing the number of casualties that are able to simultaneously re-robe from five through to 10 brings the average time in the system down to less than 13 minutes. Further increasing the re-robe capacity has diminishing benefits with a re-robe capacity of 14 casualties achieving the lowest average time in the system of 12.6 minutes. This limit occurs because the re-robe section at larger capacities no longer causes a bottleneck in the system.

If we instead consider the worst-case re-robe distribution then the average simulated time in the MD1 is 27.6 minutes (Figure 5). Because re-robing takes far longer here substantial reductions in the average time in the system continue to be made with re-robe capacity increases of 10 though to 15 casualties. The limiting lowest average time in the system of 15.8 minutes is only achieved at a re-robe capacity of 20 casualties. If we further assume that the exit light of the re-robe section is removed and that casualties leave the system when they have completed re-robing (rather than during a green light) then the average time in the system can be reduced by a further 1.3 minutes regardless of re-robe capacity. This result also holds with the baseline re-robe distribution.

**Figure 6:**
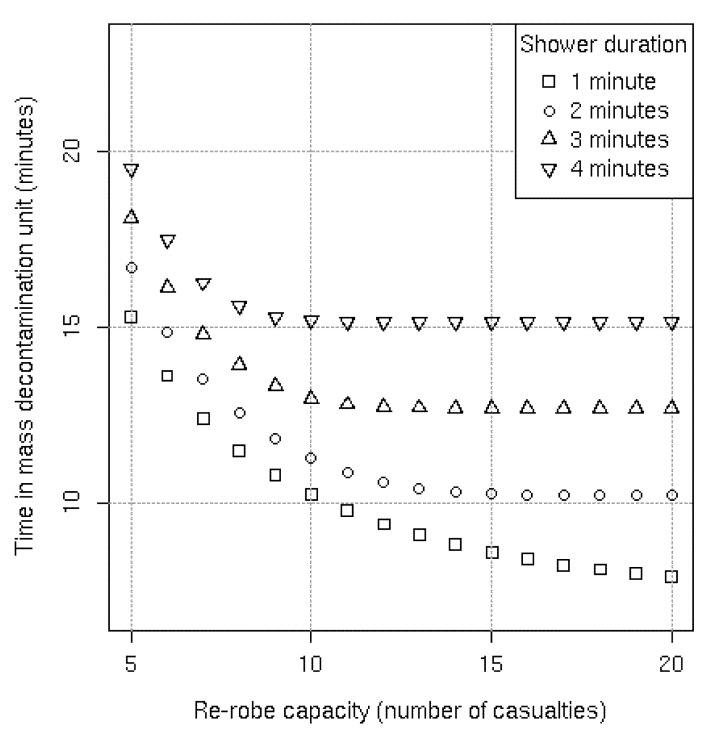
Average simulated results per lane with the baseline re-robe distribution and the exit light included.

A similar pattern emerges when instead of increasing the time required to re-robe, the showering (and final disrobing) times are decreased. Figure 6 shows that to get the greatest benefit from decreasing the showering time, the re-robe capacity must also increase. For example, with a one minute shower, reductions in the average time in the system continue to be achieved with a re-robe capacity of 20 casualties compared to a capacity of 10 casualties with a three minute shower. This occurs because the rate at which casualties move into the re-robe section is far greater, thus requiring additional capacity to mitigate the bottleneck. Figure 6 also shows how the current system would only achieve a 1.5 minute reduction for every one minute reduction in shower compared with a 2.5 minute reduction with a sufficiently large re-robe capacity.

The number of simulated casualties present in the re-robe section with an unlimited capacity is equivalent to considering one lane of the actual MD1 with casualties “cramming” into the current re-robe section. Table 1 shows that with the baseline re-robe distribution on average there could be seven casualties simultaneously re-robing if the exit light to the re-robe section was removed or the shower duration was increased to four minutes. Although this is only two casualties higher than the current re-robe capacity it should be noted that under these circumstances having 10 or 11 casualties simultaneously re-robing might not be uncommon. Even more striking is that by decreasing the shower duration to one minute, there could be 19 casualties on average in the current re-robe section and it might be rare to have fewer than 15 casualties trying to simultaneously re-robe (see Table 1).

**Table 1:** The number of casualties present in one lane of the re-robe section (utilisation) with an unlimited re-robe capacity and with different parameter values.

Re-robe parameters			Re-robe utilisation		
			(number of casualties)		
Distribution	Traffic light	Shower duration (minutes)	2.5% ile	Median	97.5% ile
Baseline	Yes	3	6	9	12
Baseline	No	3	3	7	11
Baseline	Yes	1	15	19	24
Baseline	Yes	2	9	12	16
Baseline	Yes	4	5	7	10
Worst-case	Yes	3	11	14	19
Worst-case	No	3	8	12	17

## 6. Discussion

Personal communications between the authors and FRS personnel suggests that the issues with the re-robe section highlighted in this study are well known. On three separate occasions un-related to the field exercises evaluated in this study, re-robing has been described as often taking longer than the allotted three minutes and that casualties are sometimes stopped from entering the MD1 to allow the bottleneck in the re-robe section to ease. Although this solution is relatively simple to implement it does not exploit the potential of the system and falls short of decontaminating the theoretical maximum of 180 casualties per hour per MD1. Our proposal of increasing the re-robe capacity has also been mentioned by FRS personnel during previous discussions; however, the results in this study provide a scientific platform on which to base decisions regarding the most appropriate capacity. Given that a per lane re-robe capacity of 10 effectively minimises the average time in the system with the baseline re-robe distribution (reflecting a population consisting mainly of able-bodied casualties) perhaps the most practical solution would be to provide a second re-robe section making the MD1 four sections in total. With the worst-case re-robe distribution (reflecting a population consisting mainly of disabled casualties) or if the shower duration was decreased without a detrimental effect on the efficacy of decontamination then consideration might have to be given to a larger or additional re-robe section/s. For example, the process of re-robing might be split in two, analogous to the initial/final disrobe areas; the drying process could potentially be performed in the “initial re-robe” section, with the dressing occurring in the “final re-robe” section. Such proposals, if deemed practical, would have to be tested in future exercises with further data collection of the dry/dress timings allowing informed extensions to the model described in this study.

Immediately after the Tokyo sarin attack there was no field decontamination of casualties on site; instead the local fire department established an emergency rescue quarter at the affected stations containing extra-large ambulances equipped with eight beds and large tents expandable with compressed air. Triage tags were available but were not used because the vast majority of casualties went to local hospitals on foot or via taxis [1]. In the aftermath of the Bhopal disaster there was no mass casualty emergency response system in place and local hospitals were soon overwhelmed with casualties [5]. Even with the UK’s current capability of implementing mass decontamination procedures at a safe distance from the Hot Zone it is still likely that many casualties will quickly leave the scene and self-present at hospitals largely in the following six hours [16]. This brings into question our assumption that ambulant casualties having performed their initial disrobe (averaging approximately 10 minutes, see Figure A8 in the Supplementary Material) will be queuing for the MD1 prior to its construction. Indeed, we have observed a trickle of casualties into the MD1 (and even then a subsequent overloading of the re-robe section—see Figure A4 in the Supplementary Material) during the two field exercises reported here. However, our assumption that casualties will be ready to enter the final disrobe section in groups of five (fe)males per lane provides a rigorous test of the system as it is designed. In addition, previous studies have highlighted the uncertainty in arrival times following mass casualty incidents [8,9], making it difficult to quantify and justify different numbers of casualties entering simultaneously.

Although we collected additional casualty movement data relating to the varying stages of the emergency response outside of the MD1 during both field exercises (see Figure 1 and the Supplementary Material), it is difficult to gauge how such information might help to inform the modelling of a more complete system. For example, as casualties left the re-robe section in Exercise 1 they quickly entered a casualty clearing station with a capacity of 12 whereas in Exercise 2 the casualties were transported in a mini-bus to a survivor centre with an essentially unlimited capacity. During such field exercises the experiences of casualties can be captured via questionnaires and interviews providing useful feedback for emergency response organisations. However, from a modelling perspective the data might be considered exercise dependent and not generally applicable—Albores and Shaw summarise the predicament as follows: “…one limitation is the availability of accurate input data—a result of rare, unexpected incidents being heavily effected by uncontrollable variables. Evaluations of real incidents, and large-scale simulation [field] exercises, may provide this data although such simulations tend to lack the chaos and confusion of unprepared members of the public (and some staff) which permeate real disasters and which compromises the quality of the data” [10]. Nevertheless, the MD1 is one component of the entire process that has been distributed nationally and where data collection and modelling can provide useful insights.

## 7. Conclusions

The two main objectives of this study were to collect casualty movement data from two mass decontamination field exercises and to use these data to inform a computer model of the mass decontamination process. The data collected during the exercises, supported by observations, suggest that the current flow control system of MD1 mass decontamination units might be enhanced by staggering the timings of the red/green lights at the entrance and exit of each internal section. Outputs from the computer simulations suggest that consideration should be given to at least one additional re-robe section per MD1 in order to increase the re-robe capacity and maximise the throughput of casualties. It is clear that effective communication between emergency services personnel and casualties is at least as important as any of the potential system enhancements outlined above.

## Figures and Tables

**Figure A1:**
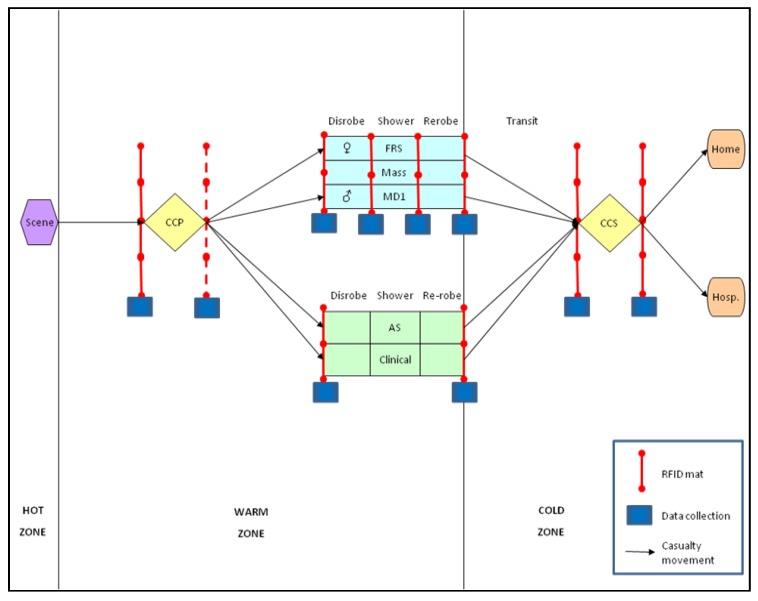
For the purposes of this analysis the warm zone extended to the exits of both the MD1 and clinical decontamination units. The casualty collection point (CCP) extended to the entrance of both decontamination units making the dashed radio frequency identification (RFID) detection mat redundant. The outer lanes of the MD1 were used to process (fe)male ambulant casualties whereas the middle lane was for non-ambulant casualties if required but only when specifically configured and not simultaneously with mass decontamination. Human evaluators captured the disrobe, shower and re-robe timings within the clinical decontamination unit. The exit to the casualty clearing station (CCS) was considered the exit from the system although the police did perform subsequent interviews.

**Figure A2:**
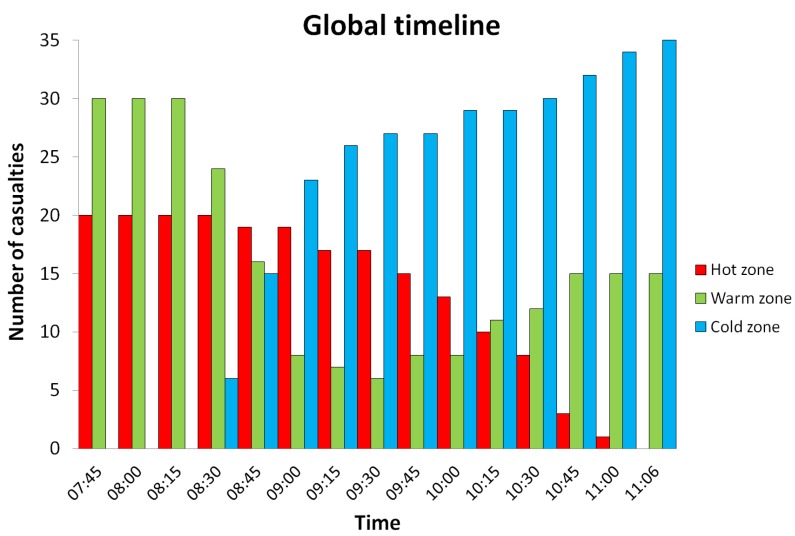
There were a total of 50 casualties (29 males, 21 females) in the system but due to the first 45 minutes ‘notional play’ the exercise started with 20 casualties (12 males, 8 females) in the hot zone and 30 casualties (17 males, 13 females) in the warm zone. There was no casualty movement between the hot and warm zones or the warm and cold zones in the first 30 minutes of the exercise. The “V” shape of the warm zone graph is a result of the flow of ambulant casualties through to the cold zone followed by the bottle-neck of non-ambulant casualties in the warm zone. At the end of the exercise (total duration 3 hours 21 minutes) all 20 casualties had been removed from the hot zone, 15 casualties (10 males, 5 females) were in the warm zone and 35 casualties (19 males, 16 females) were in the cold zone.

**Figure A3:**
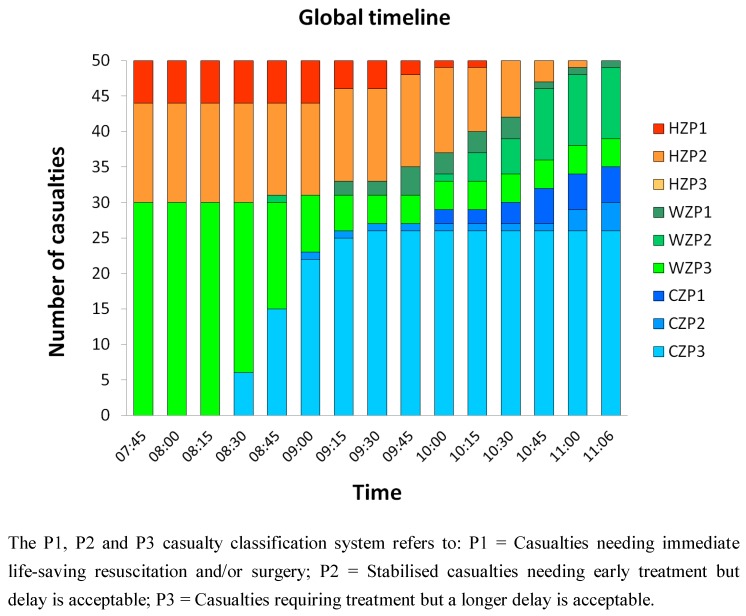
This graph represents the same information as in Figure A2 but with the data stratified by severity of injury (*i.e.*, P1, P2, and P3). Due to the first 45 minutes “notional play” there were no P3 casualties in the hot zone at the beginning of the exercise. The first P2 casualty entered the cold zone after 1 hour (having walked unaided from the hot zone to the warm zone) and the first P1 casualty entered the cold zone after 2 hours. The final P1 casualty was removed from the hot zone after 2 hours 30 minutes and the final P2 casualty was removed from the hot zone after 3 hours 15 minutes. The four P3 casualties that were still in the warm zone at the end of the exercise were those with non-waterproof electronic prosthetic legs.

**Figure A4:**
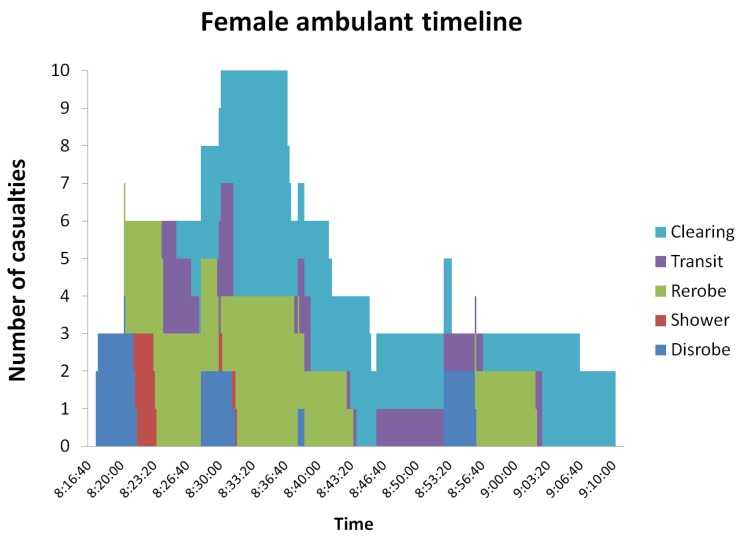
13 female ambulant casualties passed through the *disrobe*, *shower* and *re-robe* sections of the MD1 before a *transit* to the casualty *clearing* station. The times on the x-axis correspond to the approximate timing of the start of the flow control system’s green light; the graph represents 10 second time slices. Only 1 group of 3 casualties spent more than 30 seconds in the shower but this group didn’t stay in for the full 3 minute duration. For a period of approximately 30 seconds the re-robe section contained 6 casualties, 1 greater than the intended capacity of 5. For a number of casualties a significant amount of time was taken in transit, a distance of approximately 30 metres.

**Figure A5:**
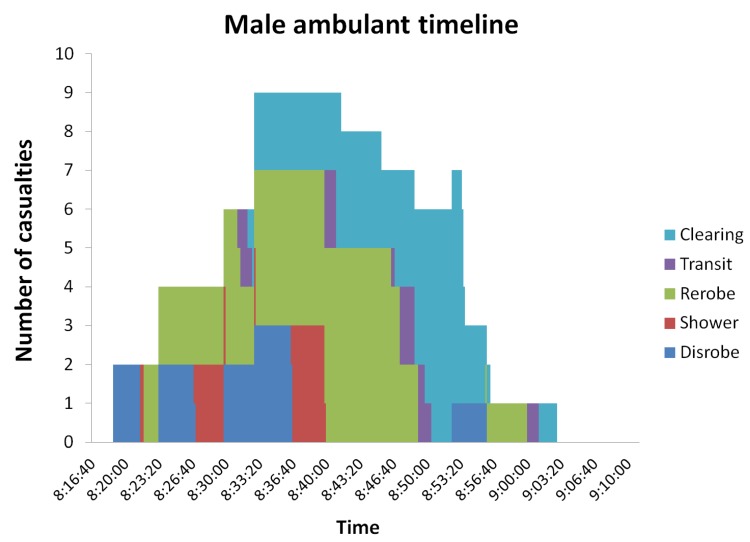
11 male ambulant casualties passed through as described in the caption of Figure A4 but 1 of these casualties was untracked through the entire process. 1 group of 2 casualties and 1 group of 3 casualties were in the shower section for the full 3 minute duration. The re-robe section was at the intended capacity of 5 for approximately 6 minutes despite groups of less than 5 entering the MD1. The casualty clearing station never exceeded the intended capacity of 12 when combining both male and female casualties. The final male casualty to go through the system was a member of the film crew having re-entered the hot zone; this was also the case for the two final females in Figure A4.

**Figure A6:**
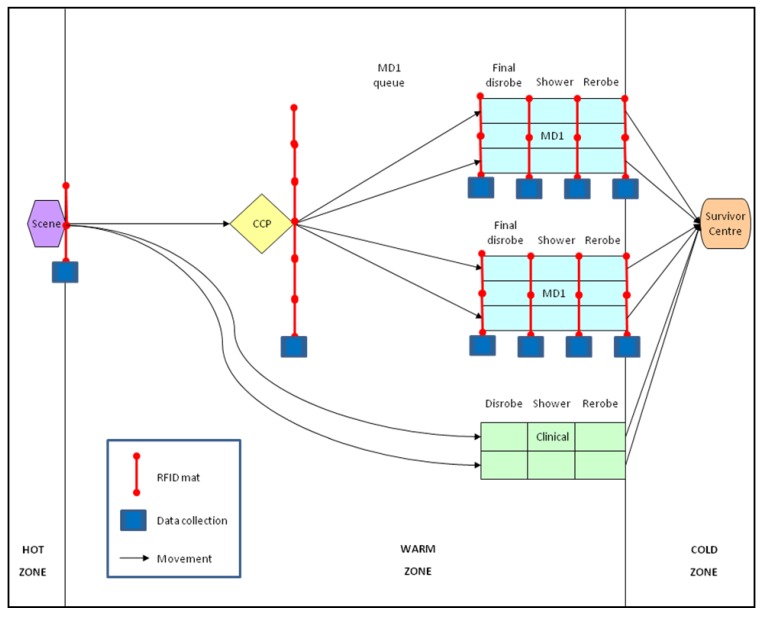
For the purposes of this analysis the warm zone extended to the exits of the three decontamination units (2 × MD1, 1 × Clinical). The initial disrobe was actually performed on the line of 6 radio frequency identification (RFID) detection mats at the edge of the casualty collection point (CCP).The outer lanes of the MD1 were used to process (fe)male ambulant casualties whereas the middle lane was for non-ambulant casualties if required but only when specifically configured and not simultaneously with mass decontamination. Human evaluators captured the disrobe, shower and re-robe timings within the clinical decontamination unit. The exits from the three decontamination units were considered the exits from the system although casualty clearing notionally took place at the survivor centre.

**Figure A7:**
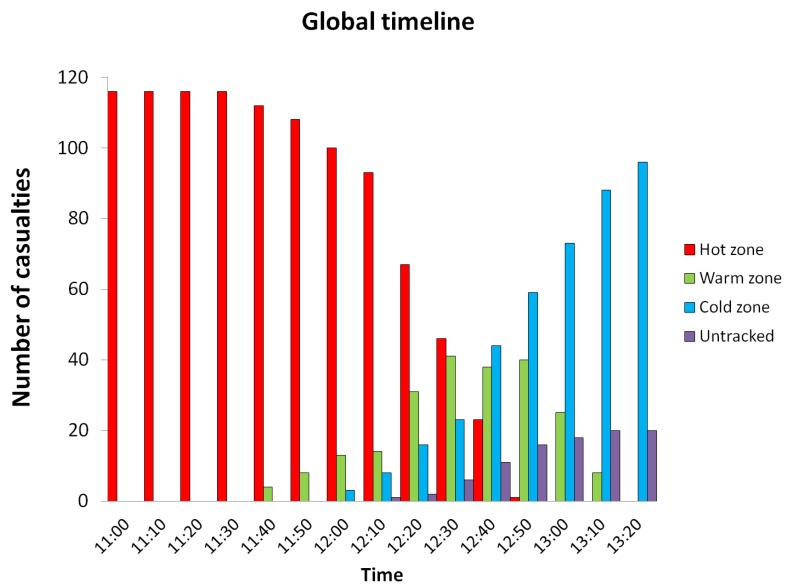
There were 130 casualties (92 males, 38 females) but 14 casualties (4 males, 10 females) were instructed to by-pass decontamination by exercise players leaving a total of 116 casualties (88 males, 28 females) in the system. 20 ambulant casualties (13 males, 7 females) removed their tags at the initial disrobe following player instruction meaning that they were untracked through the MD1s. There was no casualty movement between the hot and warm zones in the first 30 minutes of the exercise despite the decontamination units being ready to process casualties. The “Λ” shape of the warm zone graph is primarily a result of the increasing numbers of casualties that performed their initial disrobe followed by their subsequent decontamination. It took 2 hours 11 minutes to process 116 casualties through the decontamination process although it is impossible to know exactly when the untracked casualties were processed.

**Figure A8:**
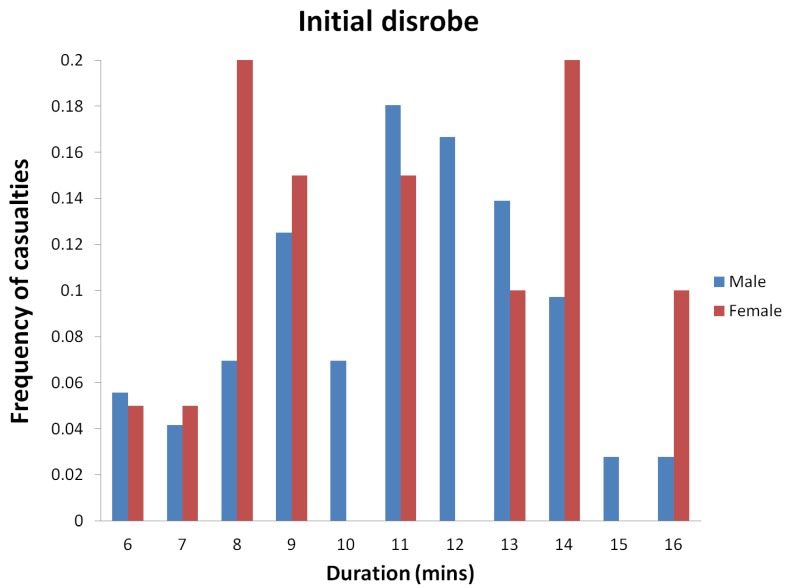
The time taken to perform the initial disrobe at the casualty collection point was captured for 72 males and 20 females. To complete their initial disrobe both males and females took on average 10.5 minutes, and no males nor females took less than 5 minutes or more than 16 minutes.

**Figure A9:**
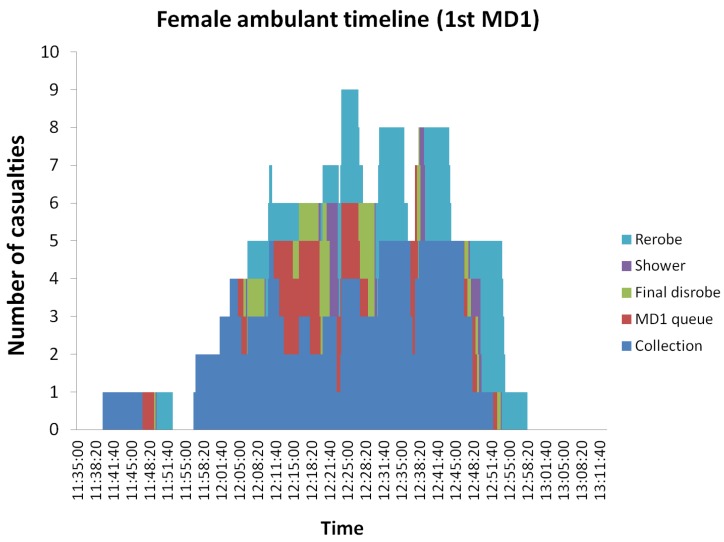
18 female ambulant casualties passed through the casualty *collection* point where they performed their initial disrobe, and then waited in the *MD1 queue *before entering the *final disrobe, shower* and *re-robe* sections of the MD1. 10 different groups entered the MD1 giving an average group size of 1.8 (range = {1,3}). 10 casualties (56%) spent less than 30 seconds in the shower. The re-robe section was briefly at the intended capacity of 5 despite the shower section having at most 3 casualties at any one time.

**Figure A10:**
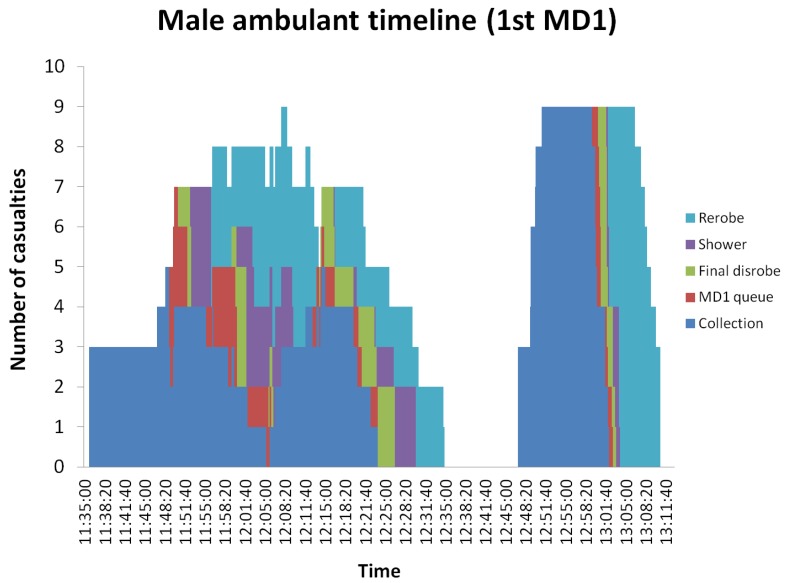
25 male ambulant casualties passed through as described in the caption of Figure A9. 11 different groups entered the MD1 giving an average group size of 2.3 (range = {1,5}). 9 casualties (36%) spent less than 30 seconds in the shower. For a period of approximately 3 minutes the re-robe section contained 9 casualties, 4 greater than the intended capacity of 5. At approximately 12:30 the flow control system stopped working which may have resulted in the approximate 20 minute time gap in processing casualties.

**Figure A11:**
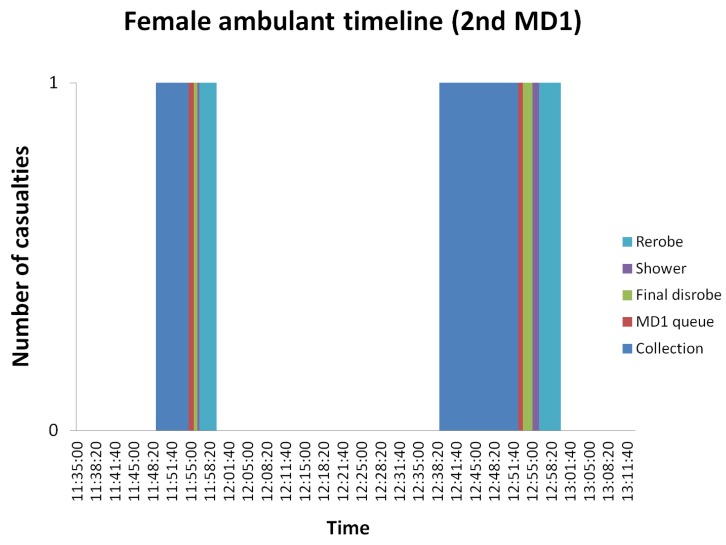
Only 2 female ambulant casualties passed through as described in the caption of Figure A9 both of whom spent more time in the re-robe section than in the final disrobe and shower sections combined.

**Figure A12:**
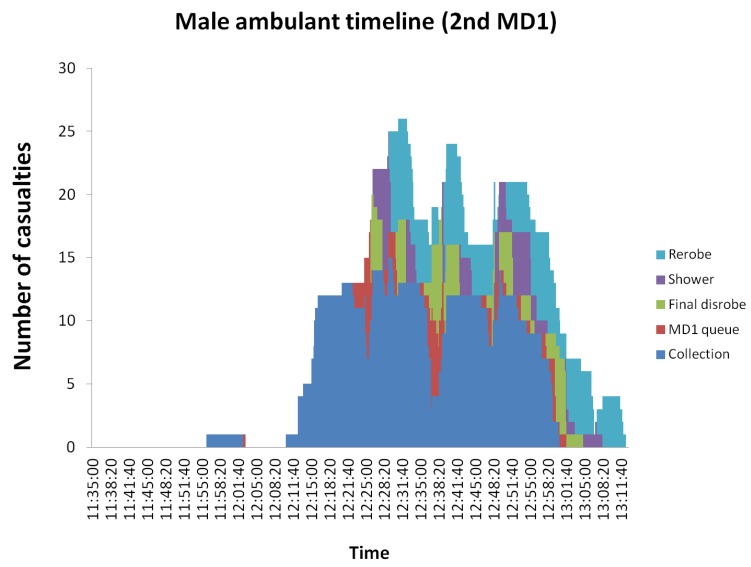
In contrast to the female ambulant timeline in Figure A11, 47 male ambulant casualties passed through as described in the caption of Figure A9. 13 different groups entered the MD1 giving an average group size of 3.6 (range = {1,8}). 14 casualties (32% as 3 casualties were untracked) spent less than 30 seconds in the shower. At times there were 8 casualties in the final disrobe, shower and re-robe sections, 3 more than the intended capacity of 5 casualties per section.

## Appendix

### Field Exercise 1. Casualty Movement Analysis.

Schematic:

All casualties:

Ambulant casualties:

### Field Exercise 2. Casualty Movement Analysis.

Schematic:

All casualties:

Ambulant casualties:
